# Metallic ions as therapeutic agents in tissue engineering scaffolds: an overview of their biological applications and strategies for new developments

**DOI:** 10.1098/rsif.2011.0611

**Published:** 2011-12-07

**Authors:** Viviana Mouriño, Juan Pablo Cattalini, Aldo R. Boccaccini

**Affiliations:** 1 Department of Pharmaceutical Technology, Faculty of Pharmacy and Biochemistry, University of Buenos Aires, 956 Junín Street, Sixth Floor, Buenos Aires CP1113, Argentina; 2 CONICET, 1917 Rivadavia Avenue, Buenos Aires CP C1033AAJ, Argentina; 3 Department of Materials Science and Engineering, Institute of Biomaterials, University of Erlangen-Nuremberg, 91058 Erlangen, Germany

**Keywords:** metallic ions, tissue engineering, scaffolds, bone, drug delivery, controlled release

## Abstract

This article provides an overview on the application of metallic ions in the fields of regenerative medicine and tissue engineering, focusing on their therapeutic applications and the need to design strategies for controlling the release of loaded ions from biomaterial scaffolds. A detailed summary of relevant metallic ions with potential use in tissue engineering approaches is presented. Remaining challenges in the field and directions for future research efforts with focus on the key variables needed to be taken into account when considering the controlled release of metallic ions in tissue engineering therapeutics are also highlighted.

## 1. Introduction

A common tissue engineering approach involves the development of novel biomaterials to produce three-dimensional porous scaffolds, that encourage cell infiltration and proliferation for tissue regeneration [1–3]. Several debilitating and deadly conditions such as osteoporosis, osteoarthritis, retinopathy, burns, myocardial infarction as well as tendon and ligament defects, among others have the potential to be treated by tissue engineering strategies. Scaffolds made from biodegradable polymers, ceramics or their composites are popular choices for tissue engineering applications and there are increasing investigations focusing on loading engineered scaffolds with therapeutic drugs, generating a dual function for the matrices: scaffolds for the growth of new tissue and carriers for controlled *in situ* drug delivery [4–13]. In addition, there is growing interest in developing matrices with the capacity to induce specific interactions within cells in order to unlock the innate path for self-repair [14]. Further, it is worthwhile noting the current expansion of the field of therapeutic tissue engineering (TTE), which considers the enhancement of the functionality of scaffolds by incorporating a drug delivery function with therapeutic effectiveness [12,15]. In this context, in order to design and develop TTE scaffolds, several variables have to be taken into account. From a tissue engineering perspective, selection of suitable processing methods that provide the best mechanical and structural properties to the final porous scaffold is of highest relevance [16]. From a pharmaceutical perspective, the scaffold fabrication method must be compatible with drug stability and sustained drug release; conditions such as high temperature, use of some organic solvents, pressure and free radicals that may lead to drug decomposition will restrict the selection of fabrication processes [17]. There are several otherwise very convenient fabrication techniques for tissue engineering scaffolds, involving however, processes that are incompatible with the incorporation and stability of organic drugs [12]. It is therefore attractive to explore the use of metallic ions as therapeutic agents (MITAs) within the scope of TTE. A wide range of MITA, the majority of them being essential cofactors of enzymes, can be considered in this regard, including cobalt, copper, gallium, iron, manganese, silver, strontium, vanadium and zinc, and will be discussed further in this article. The use of MITA does not pose the risk of decomposition or instability, which is intrinsic to organic molecules. Further, the unique properties of MITA with therapeutic significance (e.g. hydrolytic and redox activity, Lewis acidity, electrophilicity, valency, geometry, magnetic effect, spectroscopy, radiochemical properties) indicate the ability of these ions to interact with other ions that can alter cellular functions, cell metabolism or biological functions, by binding to macromolecules such as enzymes and nucleic acids, and/or activating ion channels or secondary signalling [17]. These actions of MITA may provide effects that are different from those that can be achieved through other chemical, biochemical or genetic manipulations [17]. In addition, an MITA approach is usually economic and stable under typical processing conditions for biomaterial scaffold production, which may involve the use of organic solvents, high temperatures, pressure and free radicals. Nevertheless, the potential toxicity of metallic ions when delivered locally has to be taken into account. From this perspective, the purpose of this review is to provide an overview of the advances in the expanding field of application of metallic ions in regenerative medicine and tissue engineering, focusing on their therapeutic applications. Particular emphasis is given to bone tissue engineering (TE), as this particular TE area seems to be the more developed regarding the use of MITA (also named bioinorganics [18]). This article is not encyclopaedic; rather, selected examples have been chosen to illustrate and summarize the progress in the research field. In addition, some works that detail the use of MITA to regulate specific metabolic processes are included despite not yet being used in tissue engineering, but with the potential to be considered in future TE strategies. The article is organized in the following manner: §2 discusses the general local release of metallic ions and their interaction with metabolic processes, §3 focuses on the key variables needed to be taken into account when considering the inclusion of MITA in controlled drug delivery systems in general and in scaffolds for tissue engineering in particular. Finally, remaining challenges in the field and directions for future research efforts are highlighted in §4.

## 2. Localized release of metallic ions

In the body, various metallic ions act as cofactors of enzymes and stimulate a chain of reactions associated with cell signalling pathways towards tissue equilibrium [19]. These properties, far from specific, are reflected in the very wide range of pathological conditions in which metallic ions are involved. Interactions with metallic ions play important roles in a variety of diseases and metabolic disorders such as cancer, central nervous system disorders, infectious diseases, perturbation of gastrointestinal activity and endocrine disorders; studies based on the effects of metallic ions in a wide range of pathologies are reviewed in the literature [17,20]. Thus, the efficiency and selectivity of the therapeutic effect of metallic ions can be improved by controlling the precise level and/or location of them in the body. In addition, the ionic states of certain metallic ions are unstable, and they may have toxic effects when directly ingested. To overcome these disadvantages, extensive research has been conducted to develop matrices to control the local release of metallic ions. Current metallic-ion-based drugs are prone to lead to significant systemic toxicity; thus, the design of matrices for the local delivery of relatively high concentrations of metallic-ion-based drugs to target tissues with reduced systemic adverse effects is of high interest. The degree of metallic ion loading into matrices for local delivery and the controlled and sustained release of the loaded ions are undoubtedly important to ultimately optimize metal ion delivery for therapeutic use. In addition, it is imperative to control the release rate of loaded ions. Nevertheless, ascertaining the appropriate degree of metallic ion loading and the appropriate amount released in a determined period is difficult because therapeutic levels of most MITA are unknown. A strategy very often used to load metallic ions into matrices is to bind them to a suitable substrate (zeolites, hydroxyapatite, bioactive glass, silica, carbon fibres) so that the stability of ionic states is improved and the ions can be released over a long period of time with potential applications in many fields [21–32]. Despite the efforts made in this respect, the achievements in controlling and sustaining the release of loaded therapeutic metal ions—in terms of obtaining constant therapeutic amount release of the ion over a period of time—have been very limited [18]. Amorphous peroxititanates (APT) might also be used to bind a variety of metal compounds with high-affinity forming complexes to control the delivery of metal-based drugs to the target tissue avoiding systemic toxicity, or to capture metal ions from body tissues [33–37]. Wataha *et al*. [33] demonstrated that metal–APT complexes facilitate metal ion delivery (such as gold and platinum) to monocytes as well as fibroblasts. Despite the improvements made in controlling ion release from a variety of biomaterials, potential accumulation and toxicity require further research. In healthy systems, free metallic ion concentrations are maintained at very low levels, and the normal metal metabolism delivers them in a selective manner to their sites of action, while maintaining rigid control over their reactivity. However, anomalous metallic ion metabolism can contribute to pathological states such as haemochromatosis, Wilson disease and Menkes disease [38–40]. Moreover, as mentioned above, the singular properties of metallic ions, such as Lewis acidity, hydrolytic and redox activity, electrophilicity and valency, can alter cellular activities supporting the cell metabolism or, in the worse case scenario, inducing toxic effects. For example, minimal shortages of certain metallic ions are involved in the pathogenesis of various chronic diseases such as diabetes mellitus, rheumatoid arthritis, coronary heart disease, epilepsy, nephropathy and a variety of bone-related pathologies [41–45]. By contrast, the uncontrolled release of metal ions may produce adverse effects such as the case of corrosion of metal implants, which causes the release of significant amounts of metal ions into the tissues in close contact with the implant and the systemic circulation, often resulting in complications such as inflammatory and immune reactions [46–48]. The actions of MITA loaded within matrices for local release in general, and within scaffolds for tissue engineering in particular, may be different from those that can be achieved through other chemical, biochemical or genetic interactions. The local actions of MITA within the environment in which they are released are presumed to differ in general from the actions of non-metallic agents, offering singular therapeutic opportunities. On the other hand, it is important to gain control over the potential toxicity of MITA, and the appropriate therapeutic concentrations for local release must be defined. In this context and considering the growing interest in the local release of metallic ions for therapeutic purposes, the following issues must be taken into consideration: (i) reactions of metallic ions with cellular constituents (e.g. proteins, nucleic acids—DNA and RNA—lipids, carbohydrates, redox substrates, signalling molecules); (ii) reactions of metallic ions in the local cellular environment; (iii) incorporation of metallic ions into cells and delivery to specific organelles and cellular structures; and (iv) interactions of metallic ions with specific receptors and enzymes and their involvement in metabolic pathways to alter cell functions [49,50].

## 3. Therapeutic ions in tissue engineering

**Figure 1.**
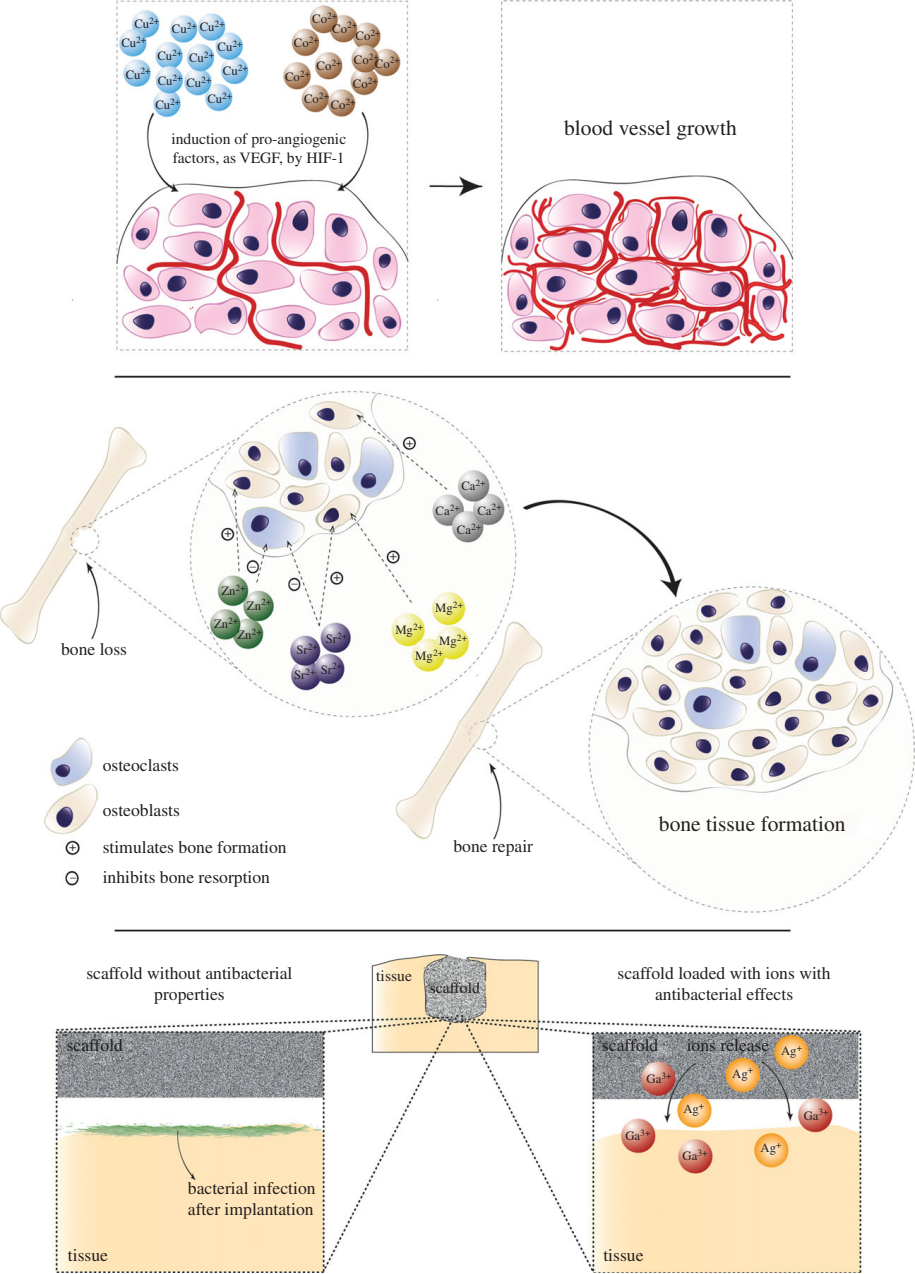
Most common specific targets of relevant metallic ions in their role of therapeutic agents. VEGF, vascular endothelial growth factor.

The interest in the application of MITA in the field of regenerative medicine and TE scaffold development is growing owing to the fact that MITA may offer therapeutic opportunities coupled with high flexibility to be incorporated in engineered biomaterial scaffolds by a broad range of processing methods. Moreover, MITA have lower cost, higher stability and potentially greater safety than recombinant proteins or genetic engineering approaches [50]. Table 1 summarizes the most common processes reported to produce scaffolds for tissue engineering with the potential to be used for the incorporation of MITA in scaffolds. Processing techniques such as rapid prototyping, electrospinning, thermally induced phase separation and solid free form fabrication are attractive because they enable fabrication of engineered three-dimensional, porous structures of high uniformity and reproducibility [81–89]. Additionally, organic/inorganic composite scaffolds, particularly for bone tissue engineering applications, made of bioceramics or bioactive glasses and biodegradable polymers [16], often include metallic ions as part of the bioceramic or bioactive glass structural composition. These inorganic materials enable metal ion release during their degradation *in vitro* or *in vivo* [18,32]. For example, when bioactive glass (e.g. 45S5 Bioglass) [90] is used in scaffolds for bone tissue engineering and introduced to fill a bone defect, critical concentrations of soluble Si, Ca, P and Na ions are released, with the capability to produce both intracellular and extracellular effects at the interface between the glass and the cellular environment [32,60,91–101]. These ions are known to stimulate various processes; for example, several investigations have demonstrated that released ions from bioactive glasses are able to induce gene expression with known roles in processes related to bone metabolism by signal transduction, thereby enhancing cell differentiation and osteogenesis [91,94,95,102]. The ionic dissolution products of bioactive glasses can also promote angiogenesis [103]. It is, therefore, vital that the kinetics of ion release from any scaffold (or implant) made from bioceramics can be tailored and controlled [18]. A comprehensive review about the biological response to ionic dissolution products from glass–ceramics and bioactive glasses in the context of bone tissue engineering has been recently published [32]. Nevertheless, it is important to highlight that few studies are focused on developing ideal matrices for the control and sustained release of loaded ions within specific therapeutic levels, over a previously defined period of time. Several attempts to intentionally load therapeutic metal ions rely on ion substitution in ceramic systems, limiting the possibility to control and sustain the release of a specific therapeutic dose over a period of time. In this sense, the application of inorganic ions in the field of bone regeneration, with special emphasis on the lack of a controlled, sustained and localized release of both structural and non-structural ions from bioceramics, is discussed in a recent report of Habibovic & Barralet [18]. Novel strategies are based on biodegradable metals, such as magnesium alloys and iron, which are dissolved *in vivo* when no longer needed [104]. In this regard, research activities are underway to make biodegradable metals practical for tissue engineering [105]. There are also new techniques to produce degradable metallic implants, innovative coating technologies to yield special surface functionalities, new biodegradable materials and methods to develop nano-devices for monitoring implants and sensing functions [104,106–113]. In addition, the use of metallic ions as cross-linkers of polymers in the formation of hydrogels and as network formers or modifiers of bioactive glasses (silicate or phosphate systems) in the elaboration of bioactive scaffolds are being increasingly investigated [13,32,51,114–118]. Figure 1 provides a summary of the most common specific targets of relevant metallic ions reviewed in the present work in their role as therapeutic agents. Table 2 summarizes relevant functions and biological effects of metallic ions with promising applications in tissue engineering. As indicated above, one of the obvious negative effects of the localized release of ions could be potential ion accumulation and toxicity. It is, therefore, vital that the kinetic of ion release from any scaffold is tailored. Several investigations have shown how the incorporation of specific metallic ions in different matrices could affect (usually improve) the physiology and metabolism of cells close to the release area; a summary of previous investigations is presented in table 3. The list is intended to be illustrative, not exhaustive. The number of specific investigations on effects of MITA intentionally added to scaffolds for therapeutic purposes, aimed at engineering a wide range of tissues, is continuously growing. Particularly, in the case of bone TE, there is increasing interest in the role of certain metallic ions (e.g. copper, strontium and zinc) in bone pathologic states because many of them are cofactors in metabolic processes involving bone, articular tissues and immune system functions [43,246]. Further, the loading of MITA within scaffolds lacks the risk of drug decomposition or instability depending on the employed processes of fabrication, as explained above. Moreover, bacterial adhesion to biomaterials that causes biomaterial-centred infection and poor tissue integration are problems that could limit the viability of the scaffold, especially when it is designed to be applied *in vivo* (as opposed to applications in bioreactors, for example) [91]. As mentioned above, there is growing interest in exploring the possibility of using the device itself to deliver therapeutic drugs to prevent possible bacterial colonization of the device following implant surgery and/or pro-angiogenic agents to secure vascularization [13,32,91,117,141]. In this context, bacteriostatic effect and pro-angiogenic potential seem to be the most common aims of the incorporation of metallic ions within scaffolds to date and the most common ions studied in this regard are copper, silver, strontium and zinc (table 3).

**Table 1.** Summary of the most common processes reported to fabricate scaffolds for tissue engineering with capabilities to include metallic ions.

technique	characteristics	reference
melt moulding+ion-exchange	melting and sintering at high temperature+introduction of ions by ion-exchange process	[51]
solvent casting	scaffolds are prepared by dissolving/suspending polymers/ceramics in presence of porogens (such as sodium chloride, sugar crystals). After pouring the mixture into a mould, solvents are removed either by evaporation or vacuum/freeze drying. Porosity is achieved by dissolving the porogens in water. Finally, the porous materials are usually lyophilized	[52,53]
freeze drying	scaffolds are prepared by dissolving/suspending polymers/ceramics in water or in an organic solvent followed by emulsification with a water phase. After pouring the mixture into a mould, solvents are removed by freeze drying and porous are obtained	[54]
liquid/liquid thermally induced separation technique	scaffolds are prepared by dissolving/suspending polymers/ceramics in a solvent that freezes below the phase separation temperature of the polymer solution. Porous materials are obtained by subsequent freeze drying	[55]
foaming	effervescent salts (ammonium bicarbonate) are used as porogens and mixed with an organic viscous solution/suspension of polymer/ceramic. After solvent evaporation, porosity is achieved by placing scaffolds into hot water or an aqueous solution of citric acid to dissolve the salts. An alternative is to use CO_2_-based gas	[53,56,57]
replica technique	scaffolds are prepared by dipping a polyurethane sponge into a slurry of proper viscosity containing ceramic particles. The impregnation step and the removal of the exceeding slurry should be tuned in order to obtain, after the sponge removal, a defect-free porous three-dimensional scaffold. Sometimes, in order to obtain mesoporous, a tensioactive may be added to the vehicle	[58,59]
sol–gel	scaffolds are prepared by dissolving metallic metal salts or metal organic compounds in a solvent where a series of hydrolysis and polymeration reactions allows the formation of a colloidal suspension (‘sol’). After casting the ‘sol’ into a mould, a wet ‘gel’ is formed. With further drying and heat treatment, the ‘gel’ is converted into dense ceramic or glass articles	[60,61]
powder compression	scaffolds are prepared by compressing polymers/ceramics using projectiles or punch and dies. The velocity of the projectile or punch and dies is adjusted to achieve powder consolidation and the desire porosity. It can be followed by sintering. An alternative is to use uniaxial and isostatic pressure	[62–65]
laser-based processing systems	scaffolds are prepared either layer by layer by photopolyermerization of a liquid (stereolithography) or sintering of powder material (selective laser syntering). In both cases, material is swept over a build platform that is lowered for each layer	[66–72]
printing-based systems	scaffolds are prepared by printing a chemical binder onto a bed of powdered material (three-dimensional printing)	[73,74]
electrospinning	the material to be electrospun is first dissolved in a suitable solvent to obtain a viscous solution. The solution is passed through a spinneret and a high voltage is used to charge the solution	[75–77]
nozzle-based systems	a thin filament of material (extruded thermoplastic polymer) that is heated through a nozzle is printed by a fused deposition modeller. Then, the mould is negative for the scaffold fabricated via fused deposition modelling	[78–80]

**Table 2.** Summary of relevant functions and biological effects of metallic ions with promising applications in tissue engineering.

ion	functions and biological effects	experimental trial	reference
calcium	approximately 99% of the body's calcium is stored in bone. Forms hydroxyapatite in combination with phosphate		
	Ca^2+^ acts as an ionic messenger. Its movements into and out of the cytoplasm serve as a signal for many cellular processes, such as exocytosis of neurotransmitter for muscle contraction. Optimal levels of intracellular Ca^2+^ may control neurite elongation and growth cone motility *in vitro*		[119–122]
	stimulation of bone cell differentiation, osteoblast proliferation, bone metabolism and its mineralization		[123–127]
	Ca^2+^ supplants Na^+^ as the ion that depolarizes the cell in the action potential in the heart's system conduction		
	increment of release of glutamate by osteoblast cells (bone mechanosensitivity)		[126]
	seven transmembrane-spanning extracellular calcium-sensing receptors in bone cells modulates the recruitment, differentiation and survival of bone cells via activation of several intracellular signalling pathways	*in vitro/in vivo*	[125]
	Ca^2+^ increases the expression of insulin-like growth factors (IGFs) that regulate human osteoblast proliferation such as IGF-1 and IGF-II		[125]
cobalt	part of vitamin B_12_ which stimulates the production of red blood cells		
	cobalt chloride can activate the hypoxia inducible factor-1 (HIF-1) in mesenchymal stem cells and subsequently activate HIF-*α* target genes including vascular endothelial growth factor (VEGF), EPO and p21		[128,129]
	hypoxia-treated bone marrow stromal cells (BMSCs) have been applied successfully to assist in re-vascularizing ischaemic or infarcted muscles in animal models	*in vivo*	[129–131]
	upregulation of the expression of pro-angiogenic growth factors (VEGF) in a variety of cells, including BMSCs		[129,132,133]
copper	stimulation of proliferation of human endothelial cells	*in vitro*	[134]
	copper–thiolate complexes are reported to be anti-inflammatory	*in vitro*	[135]
	component of super oxide dismutasa (SOD), lysyl oxidase, ceruplasmin (CP) and cytochrome *c* oxidase (COX)	*in vitro*	[135–137]
	inhibition of synthesis and modification of three-dimensional structure of DNA. Modulation of protein synthesis. Inhibition of the activity of several enzymes (such as ATPase, DNA polymerases, ribonucleotide reductase and tyrosine-specific protein phosphatase)		[138]
	modulation of proliferation and differentiation of human mesenchymal stem cells towards osteogenic lineage	*in vitro*	[139]
	facilitating the release of growth factors and cytokines from producing cells	*in vitro*	[140]
	antibacterial properties against *Staphylococcus epidermis*		[141]
	decreases the risk of ischemia in skin flaps and can induce a vascularized capsule around cross-linked hyaluronic acid-composed hydrogel	*in vivo*	[142,143]
	involvement in the activity of several transcription factors (via HIF-1 and proline hydroxylase) and bind to cell membrane releasing complex	*in vitro*	[144–146]
	induction of endothelial growth factor and enhancement of angiogenesis *in vivo.*	*in vivo*	[147–150]
	stimulation of angiogenesis in association with FGF-2	*in vitro*	[19]
gallium	alteration of plasma membrane permeability and mitochondrial functions		
	effective in the treatment of hypercalcaemia associated with tumour metastasis in bones	*in vivo*	[151]
	Ga^3+^ inhibits bone resorption and lowers concomitant elevated plasma calcium		[152–154]
	Ga^3+^ exhibits a dose-dependent antiosteoclastic effect by reducing osteoclastic resorption, differentiation and formation, inhibits bone resorption and lowers concomitant elevated plasma calcium	*in vitro*	[155]
	Ga^3+^ inhibits *Pseudomonas aeruginosa,* methicillin-resistant *Staphylococcus aureus* and *Clostridium difficile*	*in vitro*	[156,117]
iron	participation in redox reactions of metalloproteins such as cytochrome proteins, and oxygen carrier proteins such as haemoglobin and myoglobin		[157,158]
	promotion of cell attachment and differentiation of a conditionally immortal muscle precursor cell line derived from the H-2 kb-tsA58 immortomouse	*in vitro*	[141,159]
magnesium	vital for living cells owing to the interaction with phosphate ions (ATP exists in cells normally as a chelate with Mg^2+^)		
	cofactor for many enzymes (catalytic action)		
	stimulation of growth of new bone tissue and adhesion of osteoblastic cells	*in vitro/in vivo*	[160–165]
manganese	cofactor for a very broad number of enzymes (oxidoreductases, transferases, hydrolases, lyases, isomerases, ligases, lectins, integrins and glutamine synthetase). Essential in detoxification of superoxide free radicals		[166,167]
silver	antibacterial agent (Ag^+^)		
	binding to microbial DNA (preventing replication) or to sulfhydryl groups of bacteria enzymes (inhibition of cells' respiration and bounding transportation of important substances across the cells membrane and within the cells)	*in vitro/in vivo*	[59,168–177]
strontium	it is stored in the skeleton by exchanging with Ca^2+^ in the hydroxyapatite crystal lattice, preferably in new trabecular bone and with variations depending upon the skeletal site (Sr^2+^ content increases in the sequence diaphysis of the femur, lumbar vertebra and iliac crest)		[178,179]
	low doses of Sr^2+^ have been shown to stimulate bone formation. High doses have deleterious effects on bone mineralization, through reduction in calcium absorption and possibly alterations of the mineral properties		[180]
	incrementation of bone formation and reduction of bone resorption, leading to a gain in bone mass and improvement of bone mechanical properties in normal animals and humans	*in vitro/in vivo*	[179,181]
	incrementation of osteoblast differentiation and function, reduction of osteoclast differentiation and disruption of actin cytoskeleton organization		[182]
vanadium	it works by regulating specific protein phosphatases and kinases instead of insulin hormone itself or insulin receptors messengers, possibly bypassing non-functional components of the insulin signalling pathways		[41,183–190]
	could inhibit the enzyme protein tyrosine phosphatase 1B (PTP1B). The PTP1B obstructs the active site where phosphate hydrolysis of the insulin receptor occurs, thus acting as a negative regulator of insulin signalling		[190–194]
	organic compounds decreases neuropeptide Y levels in the hypothalamus and thus an increment in the insulin sensitivity in adipose tissue and a decrement in the appetite and body fat can be observed		[195]
	proliferation and differentiation of 3T3-L1 preadipocytes		[196]
	help to lower low-density Lipoprotein cholesterol levels and impede cholesterol from building up on the walls of arteries		[197]
	can promote bone and teeth mineralization		[198]
	stimulates osteoblast proliferation and differentiation, and increases mineralization of the matrix and collagen synthesis		[199–201]
	several vanadium (Va^4+^) compounds studied (such as with ascorbic acid, maltol, threalose and non-steroidals anti-inflammatory drugs such as aspirin, ibuprofen, naproxen and tolmetin) did affect osteoblast proliferation and differentiation at low doses by stimulating cell growth and inhibiting alkaline phosphatase (ALP)-associated osteoblastic differentiation		[201–205]
zinc	zinc ion (Zn^2+^; with copper) is a component of SOD		[135]
	increment of the activity of aminoacyl-tRNA synthetase		[206]
	in bone metabolism, it is associated with growth hormone (GH) or insulin-like growth factor 1 (IGF-1)		[207,208]
	after addition of zinc to tibial cultures, the relative extend of the zinc-induced DNA increase was similar to the relative extend of the zinc-induced increase in ALP activity		[209]
	facilitates neural growth		[210]
	stimulation of bone formation by enhancing osteoblast differentiation	*in vitro/in vivo*	[128,131,209,211–216]
	is considered a stimulating bone formation agent through the increase of Runt-related transcription factor 2 targeted osteoblast differentiation gene transcription		[217]
	the interaction of Zn^2+^ with high- and low-affinity sites in Na^+^ channels may modulate neuronal excitability through a concentration-dependent biphasic effect of Zn^2+^ that could activate or inhibit the sodium current		[218]
	it has anti-inflammatory effects		[219]
	inhibits bacterial growth at the surgical site and improves wound healing		[220,221]

**Table 3.** Metallic ions included in scaffolds made of different biomaterials designed for tissue engineering.

ion	scaffold composition	experimental trial		reference
calcium	osteochondral composite using type II collagen gel with hydroxyapatite (HAP) varying amount of calcium (2–4 mmol, 6–8 mmol, less than 10 mmol) with deposit on one side (two- and three-dimensional)	*in vitro*	low Ca^2+^ concentrations (2–4 mmol) promoted osteoblast proliferation. Medium Ca^2+^ concentrations (6–8 mmol) produced differentiation and extracellular matrix mineralization. Higher concentrations (greater than 10 mmol) are cytotoxic	[123]
	calcium phosphate (CaP) treatment of the surface of three-dimensional bioactive glass scaffolds	*in vitro*	three types of bioactive glass scaffolds (non-treated, thick and thin Ca–P-treated) were compared. The expression of osteopontin and alkaline phosphatase (ALP; both indices of osteogenic differentiation) were higher in the non-treated and thin Ca–P-treated scaffolds when compared with thick Ca–P-treated scaffolds. The higher release of Ca^2+^ from thick Ca–P-treated scaffold relates to the low ALP activity and may also lead to low osteopontin synthesis	[208]
	mesoporous silica xerogels (SiO_2_–CaO–P_2_O_5_) with varying amounts of calcium (0, 5, 10 and 15 wt%) by template sol–gel method	*in vitro*	small (5 wt%) and medium (10 wt%) Ca concentrations stimulated cell proliferation but only 5 wt% Ca stimulated differentiation (indicated through ALP activity) and stimulated gene expression (via ERK1/2 activation). Higher amounts of calcium (15 wt%) tended to decrease ALP stimulation levels	[122]
cobalt	dual-layered periosteum using BMSCs treated with CoCl_2_ in a type I collagen scaffold	*in vivo*	osteogenic (BMSCs-derived osteoblasts) and pro-angiogenic cells (CoCl_2_-pre-treated BMSCs) were seeded onto opposite sides of a collagen membrane. BMSCs pre-treated with CoCl_2_ increased VEGF expression near fivefold	[129]
copper	copper nanoparticles (CuNPs) concurrent with HA oligomeric cues	*in vitro*	the release of Cu ions improved recruitment and cross-linking of soluble tropoelastin precursors and facilitated their assembly into mature fibres	[222]
	three-dimensional printed macroporous bioceramic scaffolds made by brushite	*in vivo*	very low doses of Cu^2+^ (56 ng) facilitated implant vascularization, whereas a 10-fold increase in the dose enhanced wound tissue ingrowth (560 ng Cu^2+^)	[50,223]
gallium	quaternary gallium-doped phosphate-based glasses (1, 3, and 5 mol% GA_2_O_3_) using a conventional melt quenching technique	*in vitro*	the results confirmed that the net bactericidal effect against both Gram-negative (*Escherichia coli* and *Pseudomonas aeruginosa*) and Gram-positive (*Staphylococcus aureus*, methicillin-resistant *S. aureus* and *Clostridium difficile*) bacteria, was owing to Ga^3+^, and a concentration as low as 1 mol% Ga_2_O_3_ was sufficient to mount a potent antibacterial effect	[156]
	Ga-cross-linked alginate films with nano bioactive glass	*in vitro*	the controlled release of Ga^3+^ produced the proliferation of human-like osteoblast cells and an effective prophylaxis against *S. aureus*. Further studies remain to be done, but the composite films are expected to be promising candidates for bone tissue engineering applications	[13]
iron	Fe^3+^-alginate films	*in vitro*	the capability of Fe films as scaffolds for culturing normal human dermal fibroblasts (NHDF) were compared with those obtained on alginate films containing calcium ions (Ca-alginate). No adhesion of NHDF was observed on Ca-alginate but NHDF proliferated substantially on Fe-alginate. The participation of serum proteins such as vitronectin was essential for initial attachment and spreading. The investigation also showed that significantly higher amounts of vitronectin and fibronectin were adsorbed by Fe-alginate films	[114]
magnesium	glass ceramics (49.13 wt% SiO_2_-7.68 wt% CaO-43.19 wt% MgO) with varying amounts of wt% CaO by template sol–gel method	*in vitro*	Young's modulus was similar to that of cortical bone (29.73 GPa). Osteoblast cell proliferation and differentiation were stimulated	[223]
	quaternary glass system SiO_2_–CaO–P_2_O_5_–MgO (64% SiO_2_, 26% CaO, 5% MgO and 5% P_2_O_5_ in mol%) synthesized by the sol–gel technique	*in vitro*	the incorporation of a limited amount of magnesium enhanced bioactivity. The glass system facilitated the growth of human foetal osteoblastic cells (*hFOB*1.19)	[224]
manganese	Mn(II)-substituted hydroxyapatite (Mn-HA) was produced by the wet chemical method coupled with ion-exchange mechanism and displayed non-cytotoxicity to osteoblast	*in vitro*	Mn^2+^ ions increase ligand binding affinity of integrate and activate cell adhesion to HA	[225,226]
silver	SiO_2_–CaO–P_2_O_5_–Ag_2_O (3 wt% Ag_2_O in the glass)	*in vitro*	bactericidal effect on *E. coli* MG1655, *P. aeruginosa* and *S. aureus* with Ag^+^ concentrations in the range 0.05–0.20 mg ml^−1^	[227,228]
	Ag^+^ ions were introduced into three-dimensional bioactive silicate glass–ceramic scaffold surfaces through a patented ion-exchange process	*in vitro*	the control of Ag^+^ content on the scaffold surface, as well as the Ag diffusion profile throughout the ion-exchanged layer, was achieved by controlling the ion-exchange parameters (temperature, time and silver concentration in the molten bath)	[51]
	silver-doped bioactive glass (AgBG) coating on surgical sutures, which was elaborated by using a slurry-dipping process	*in vitro*	AgBG coating had a significant effect on preventing *Staphylococcus epidermidis* attachment when compared with coatings of standard 45S5 Bioglass	[229]
	Ag^+^ incorporated on surface of scaffolds based on 45s5 bioglass	*in vitro*	cellular studies with human periodontal ligament stromal cells (HPDLCs) indicated that cell attachment was supported and cell viability was maintained on silver-doped three-dimensional scaffolds comparable to the control (un-substituted) 45S5 bioglass scaffolds	[230]
strontium	porous ceramic bone substitutes, for replacing cancellous bone or as filler in the orthopaedic and dental fields	*in vitro*	Sr^2+^ released *in vitro* became constant after one week, but Ca^2+^ release was improved for SrHA compared with stoichiometric HA, owing to the higher solubility of SrHA	[231]
	sol–gel derived bioactive silicophosphate glass based on SiO_2_–CaO–SrO–P_2_O_5_ system	*in vitro*	the glass-stimulated proliferation of rat calvaria osteoblast and enhanced cell differentiation and ALP activity	[232]
	Sr-doped BG as solid discs	*in vitro*	Sr^2+^ released (in the range of 5–23 ppm) increased osteoblast metabolic activity and inhibited osteoclast differentiation. Osteoblasts proliferation and ALP activity were observed with increasing Sr^2+^ substitution. Osteoclasts adopt typical resorption morphologies	[233]
	phase-pure strontium silicate powders (SrSiO_3_) were also developed by the chemical precipitation method	*in vitro*	bioactivity of the powder was confirmed. Cell proliferation of rabbit BMSC was observed at Si concentrations of 1.87–0.12 mM and 0.12–3.75×10^−3^ mM. There was no cytotoxicity for mouse fibroblast cells, except at high ion concentrations (Si 3.75 and Sr 0.12 mM)	[234]
	Sr was incorporated into mesoporous SiO_2_ (mSr-Si) by a modified template-induced and self-assembling method	*in vitro*	Sr^2+^ and SiO_4_^4−^ ion concentrations from mSr–Si glass reached as high as 34.5 and 102 ppm, respectively. These levels were not cytotoxic to human bone mesenchymal cells but there was a slight inhibitory effect on ALP activity when the Sr^2+^ concentration was greater than 26.5 ppm; below this, level ALP activity was comparable to that of the controls	[235]
	Sr-doped bioactive glass in the SiO_2_–CaO–SrO system manufactured by the sol–gel method	*in vitro*	osteoblast differentiation was enhanced in the presence of bioactive glass particles containing 5 wt% strontium	[236]
zinc	zinc-doped hydroxyapatite	*in vitro*	improved osteoblast cell adhesion compared to undoped hydroxyapatite	[237]
	addition of zinc ions to an organoapatite coating of titanium fibres	*in vitro*	increased ALP activity compared with undoped organoapatite or uncoated titanium fibres	[130]
	disk made by sol–gel derived CaO–P_2_O_5_–SiO_2_–ZnO bioglass containing 5 mol% ZnO	*in vitro*	increased ALP activity and osteoblast counts compared to cells cultured on either polystyrene plates or the base CaO–P_2_O_5_–SiO_2_ bioglass, indicating the possible stimulating effect to cell proliferation and differentiation by the zinc-substituted bioglass	[238]
	Zn-containing phosphate-based glasses of P50C40N10	*in vitro*	glass compositions did not reduce the pH of cell culture medium to the extent that it would be harmful to osteoblast-like cells (HOB cells) cultured on the glass and the cell attachment data showed that HOB cells remained attached to the glass discs for up to 7 days in culture	[239]
	zinc-based glass polyalkenoate cements	*in vitro*	improved antibacterial efficacy *in vitro* against bacterial strains commonly associated with infection after orthopaedic surgery against (e.g. *Streptococcus mutans* and *Actinomyces iscosus*). Zn^2+^-released cements occurred in an immediate burst over the first day after synthesis *in vitro*; after a week, the release rate fell below detectable levels. According to the authors, the antibacterial activity exhibited by the cements revealed the pattern of Zn^2+^ release	[240]
	Zn addition (5 wt%) on bioactive glass scaffold (45S5)	*in vitro*	the addition of Zn reduced solubility, enhanced bioactivity and improved conditions allowing endothelial cells to grow over a 6 day period *in vitro*	[241]
	Zn addition on bioactive glass scaffold (Na_2_O, K_2_O, MgO, CaO, B_2_O_3_, TiO_2_, P_2_O_5_ and SiO_2_)	*in vitro*	the addition of zinc slowed down its degradation profile and inhibited spreading and proliferation of human adipose stem cells (hASCs). ALP activity, DNA content and osteopontin concentration of hASCs were not significantly affected by zinc addition. It was suggested that the possible stimulatory effect of the addition of zinc on hASCs proliferation and osteogenesis was not detected because the addition of zinc retarded the degradation rate of the scaffolds	[208]
multiple association of ions	varying compositions of Ca–Sr–Na–Zn–Si glass bone grafts	*in vitro*	controlled release of Zn^2+^ and Sr^2+^ (in the 3–18 ppm and 0–3500 ppm ranges, respectively) with the potentiality to allow therapeutic levels. Higher viability of mouse fibroblast cells was observed when ionic extracts of these Zn–Sr-doped glasses were applied, compared with standard bioactive glass (Novabone)	[242]
	controlled substitution and incorporation of strontium and zinc into a calcium–silicon system to form Sr–hardystonite (Sr–Ca_2_ZnSi_2_O_7_, Sr-HT)	*in vitro* and *in vivo*	Sr-HT ceramic scaffolds induced the attachment and differentiation of cells and osteoconductivity after six weeks following implantation in tibial bone defects in rats with rapid new growth of bone into the pores of the three-dimensional scaffolds. However, Sr-HT scaffolds were less mechanically resistant when compared with a calcium-zinc–silicate system ((Ca(2)ZnSi(2)O(7)) HT)	[243]
	β-TCP co-doped with monovalent (Ag^+^) and divalent (Zn^2+^ or Cu^2+^) ions (AgZn–TCP and AgCu–TCP)	*in vitro*	antibacterial activities of AgZn–tricalcium phosphate (TCP) and AgCu–TCP on *E. coli* and *S. aureus* were higher than those of Ag^+^ ions-doped *β*-TCP (Ag–TCP) and pure *β*-TCP. According to the authors, these antimicrobial activities suggested that an interaction occurred between bacteria and Ag^+^, Zn^2+^ and Cu^2+^ ions eluted from AgZn–TCP, and AgCu–TCP, and between bacteria and the free radicals generated by antibacterial agents or in bacterial cells. The appropriate relative quantities of those ions needs to be established by taking into account their antibacterial activities and possible cytotoxicities	[244]
	calcium–strontium–zinc–silicate glass	*in vitro*	synergistic therapeutic effects to improve bone health at the implant site, for example, to those patients suffering from diseases such as osteoporosis, whereas minimizing the risk of primary deep infection at the implant site owing to the established antibacterial nature of the Zn^2+^ and Sr^2+^. The novel glass grafts are capable of inducing bone growth in close apposition to the implanted particles	[245]

## 4. Concluding remarks and future steps

Metallic ions are of interest in the fields of regenerative medicine and tissue engineering owing to the possibility of exploiting their unique advantages for therapeutic applications: reduced cost, increased stability and, in terms of safety, potentially lesser risk than techniques of recombinant proteins or genetic engineering. Several biomaterial-based strategies are being designed for the controlled-localized delivery of metallic ions and the field is continuously expanding. However, many challenges remain. First, there is a need to acquire a deep understanding of the roles of specific metals in cellular regulation and cell–cell signalling in both healthy and diseased tissue when they are released locally from scaffolds, implants or other releasing devices. Second, more *in vivo* evidence confirming that metallic ions can be released locally from scaffolds without systemic toxicity and carcinogenic effects is bound to follow [247]. In addition, broader knowledge about mechanisms linking univocally the improved biological performance provided by TE scaffolds to the effect of metallic ions release is also needed. Much of the work is expected to involve collaborations, including biologists, material scientists, pharmaceutical technologists, tissue engineers and biomedical researchers. A great deal of further work is necessary but current investigations suggest that such work may be fruitful towards more effective tissue engineering strategies with improved MITA-releasing biomaterials. The final objective of this review has been thus to encourage research that bridges the areas at the interface between materials chemistry and medicine for developing new tissue engineering therapeutic strategies based on controlled metal ion release.
